# Application of cAMP-dependent catalytic subunit β (PRKACB) Low Expression in Predicting Worse Overall Survival: A Potential Therapeutic Target for Colorectal Carcinoma

**DOI:** 10.7150/jca.46156

**Published:** 2020-06-07

**Authors:** Xiaoya Yao, Weixian Hu, Jie Zhang, Chengzhi Huang, Haibi Zhao, Xueqing Yao

**Affiliations:** 1 School of Bioscience and Bioengineering, South China University of Technology, Guangzhou, Guangdong, People's Republic of China.; 2 The Second School of Clinical Medicine, Southern Medical University, Guangzhou, People's Republic of China.; 3 Department of General Surgery, Guangdong Provincial People's Hospital, Guangdong Academy of Medical Sciences, Guangzhou, Guangdong, People's Republic of China.; 4 School of Medicine, South China University of Technology, Guangzhou, Guangdong, People's Republic of China.; 5 Shantou University Medical College, Shantou, Guangdong, People's Republic of China.

**Keywords:** PRKACB, colorectal carcinoma, survival, therapeutic target

## Abstract

Low expressions of PRKACB are related to the occurrence of various human malignancies. However, the prognostic value of PRKACB expression in colorectal cancer (CRC) patients remains controversial. In this analysis, PRKACB expression in CRC tumors was evaluated across the GEO, TCGA, and Oncomine databases, and a PRKACB survival analysis was performed based on the TCGA profile. We detected PRKACB in 7 GEO series (GSE110225, GSE32323, GSE44076, GSE9348, GSE41328, GSE21510, GSE68468) and TCGA spectra (all P <0.05). A meta-analysis performed in the Oncomine database revealed that PRKACB was significantly up-regulated in neoplastic tissues compared to normal tissues (all P <0.05). A Kaplan-Meier analysis demonstrated that lower PRKACB expression in tumors was significantly associated with poorer overall survival (OS) in patients with CRC (P <0.05). A subgroup analysis showed that low expression of PRKACB correlated with poor 1-, 3-, and 5-year OS (all P <0.05). Furthermore, in males (P = 0.0083), whites (P = 0.0463), and non-mucinous adenocarcinoma patients (P = 0.0108), the down-regulation of PRKACB expression was more significant for the OS prognostic value. Conclusion: PRKACB is down-regulated in tumors and associated with worsening OS in CRC patients.

## Introduction

Colorectal cancer (CRC) is amongst the most prevalent digestive tract malignancies and it is the world's second most deadly cancer with almost 900 000 deaths recorded annually 1. Despite formidable advances in imaging techniques, surgery and multimodal therapy, the overall survival for patients with advanced CRC remains low. A study showed that the incidence of CRC in China has constantly been on the rise over the past three decades and equally predicted a further increase in the near future 2. According to the 2015 cancer statistics 3, it is estimated that about 2,376,300 new CRC cases and 191,000 CRC related deaths occurred in China, accounting for nearlyone-tenth of the global CRC burden. Even though outstanding radiological, surgical and multimodal therapeutic advances have recently been mad, the overall survival rates for patients with late stage CRC remain substantially low at ∼8-9% 4. A precise estimation of the prognosis plays an important role in the diagnostic and therapeutic management of CRC patients. Thus, identifying reliable and practical prognostic biomarkers and revealing novel CRC treatment targets is urgently required 5,6.

The protein kinase cAMP-dependent catalytic subunit β (PRKACB) encoding the cAMP-dependent protein kinase catalytic subunit β (PRKACB) is a member of the serine/threonine protein kinase family 7. It serves as a key regulatory point and is involved in cell proliferation, differentiation, regulation of apoptosis, and is closely related to physiological and pathological processes such as cell growth, gene expression, tumor proliferation and metastasis 8. Recently, the presence of the PRKACA and PRKACB fusion genes has been detected in various cancers such as bile duct cancers, fibrolamellar hepatocellular carcinoma, and pancreatic cancers, and there is a possibility that this might be implicated in the pathogenesis of cancer 9-11. Some researches declared that PRKACB mutations could cause adrenal and bile duct tumors 12,13, and that high expressions of PRKACB is related to drug resistance in patients diagnosed with breast cancer 14. Previous studies have confirmed that the c-MYC was directly activated by PRKACB, which then induced neoplasia 15,16. Conversely, another study suggested that the PRKACB might act as a tumor suppressor gene in non-small cell lung cancers 17. Nonetheless, the exact role played by PRKACB in colorectal cancer is still unknown. Thus, to evaluating the prognostic value of the PRKACB in CRC patients is fundamental.

To help elucidate the possible relationship between the PRKACB expression and CRC patient outcomes, we identified the PRKACB expression in GEO, Oncomine and TCGA databases and performed a survival analysis based on the TCGA profile, with hopes of providing useful insights into the development of colorectal cancer.

## Materials and Methods

### Data resource and Description

GEO microarray series (GSE110225, GSE32323, GSE44076, GSE9348, GSE41328, GSE21510, GSE68468) containing CRC tumor and non-tumor samples were obtained from the National Center for Biotechnology Information's (NCBI) Gene Expression Omnibus (GEO, https://www.ncbi.nlm.nih.gov/geo/). Platforms and samples of GEO series were summarized in Table 1.

All of the publicly available colorectal cancer RNA-Seq data information were downloaded from The Cancer Genome Atlas (TCGA) official website (https://cancergenome.nih.gov/) before December 17, 2019, through the GDC Data Transfer Tool 18. TCGA RNAseq data was comprised of 568 tumor samples and 44 non-tumor samples.

### Bioinformatics analysis for identifying PRKACB expression

Raw CEL files of the microarray from each GEO dataset were normalized by the quantile method of Robust Multichip Analysis (RMA) from the R affy package 19 and the normalized gene expression levels were presented as log2-transformed values by RMA. Gene expression of PRKACB was determined by tumor and non-tumor samples comparison from the R Limma package 20.

The R language version 3.6.1 edgeR package 21,22 was used to compare the mRNA expression of tumor and non-tumor samples from TCGA.

Studies comparing PRKACB between tumor and non-tumor samples in colorectal cancer were selected with a threshold of p-Value ≤ 1E-4, fold change ≥ 2 and top 10% gene rank in the Oncomine database (https://www.oncomine.org/).

### Survival analysis

The clinical data and PRKACB RNA Seq V2 data of 382 colorectal cancer patients (TCGA, Provisional) were downloaded from the cBioPortal database 23,24 (http://www.cBioPortal.org/), and the patients' information were matched with the sample ID by VLOOKUP index in EXCEL. After deleting some missing data, the PRKACB expression values were ranked from top to bottom and the median was taken as the cutoff point, these patients were then classified into a low expression group and high expression group to analyze the correlation between PRKACB expression with survival rates and clinical pathological characteristics.

### Identification of PRKACB-related genes

RNA-Seq data information of 568 tumor samples and 44 non-tumor samples were downloaded from TCGA official website and the edgeR package 21,22 was used to compare the mRNA expression of the tumor and non-tumor samples to screen for differential expression genes (DEGs). The P-value <0.05 and |logFC|>1 were chosen as cut-off criteria. The Spearman coefficients of the DEGs and PRKACB were calculated, whilst DEGs with *p*-value<0.05 were defined as PRKACB-related genes.

### KEGG/GO biological process enrichment

The Database for Annotation, Visualization, and Integrated Discovery (DAVID, http://david.ncifcrf.gov) (version 6.8) 25 is an online functional annotation tool that we applied for Gene Ontology (GO) enrichment analysis, including biological process (BP), cellular components (CC), and molecular function (MF). The DAVID database was also used to a perform pathway enrichment analysis. *P*-value <0.05 was considered as the threshold.

### Protein-protein interaction (PPI) network construction

STRING (http://string-db.org) (version 11.0) 26 is an online biological database for the prediction of known and unknown protein interaction relationships. The PRKACB-related genes were uploaded to the STRING website to analyze the interactions between those proteins. The minimum required interaction score was set to 0.400 (medium confidence) and the protein nodes undergoing no interaction with other proteins were removed. Next, the PPI pairs were inputted into the Cytoscape software (http://www.cytoscape.org) (version 3.7.1) 27 to construct a PPI network and the top 10 hub genes were identified in accordance with the Cytoscape plug-in (degrees ranking of cytoHubba).

### Gene Co-expression Network Analysis

The mRNA expression of 7 complete samples from the Colorectal Adenocarcinoma (TCGA, Provisional) database was used to conduct an analysis by the Co-expression online analysis function in the cBioPort database (http://www.cBioPortal.org/) 24. *P* value <0.05 was considered as the threshold. Genes with Spearman correlation coefficients and PRKACB expression greater than 0.9 were screened and uploaded to Cytoscape software (http://www.cytoscape.org) (version 3.7.1) 27 to draw a gene co-expression network.

## Results

### PRKACB expression comparison

**Figure 1:**
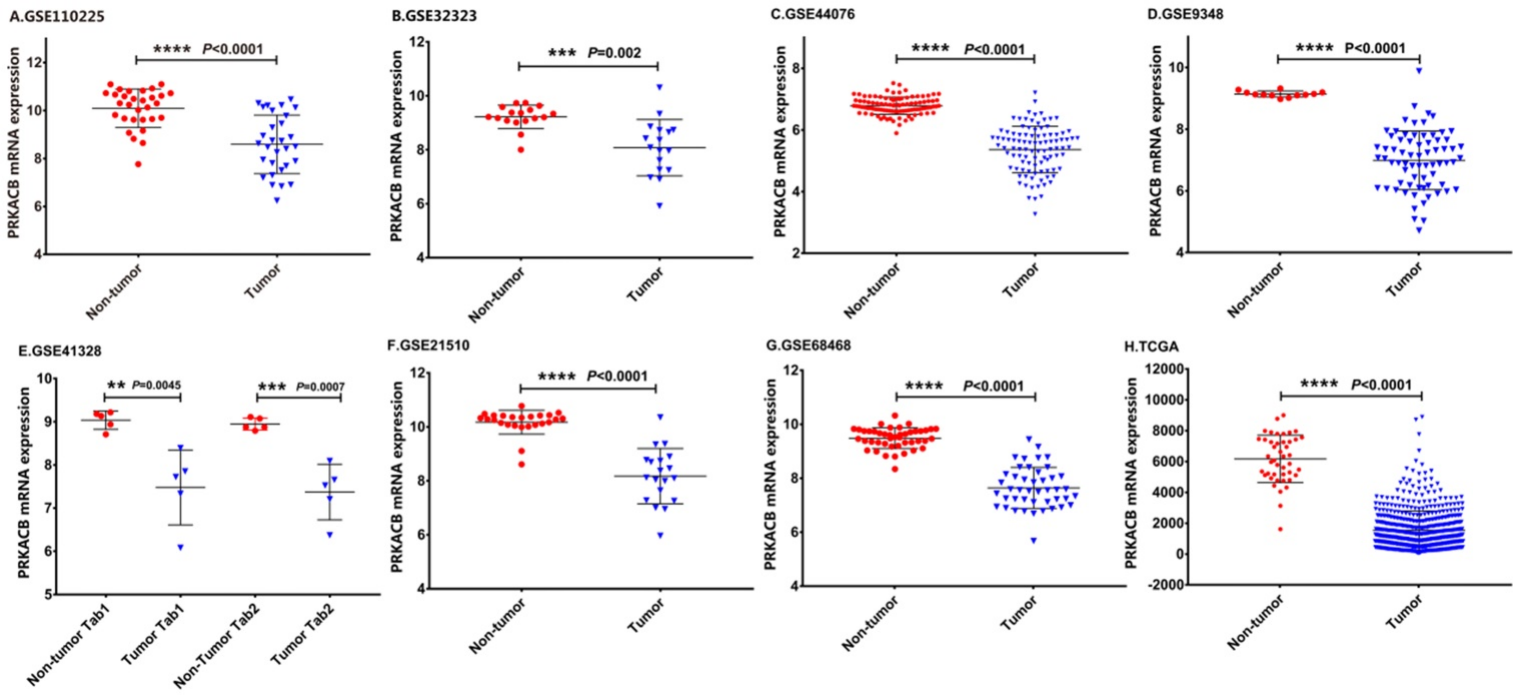
mRNA expression levels of PRKACB between tumor and non-tumor samples in CRC patients on the GEO database series including GSE110225 (A), GSE32323 (B), GSE44076 (C), GSE9348 (D), GSE4128 (E), GSE21510 (F), GSE68468 (G), TCGA database (H).

The details of the GEO series included in this analysis were summarized in Table 1. As illustrated in Figure 1, the expression of PRKACB in tumor samples was all significantly lower than non-tumor samples in GSE110225, GSE32323, GSE44076, GSE9348, GSE4128, GSE21510, GSE68468 and TCGA datasets (all P < 0.01, Figure 1).

**Figure 2:**
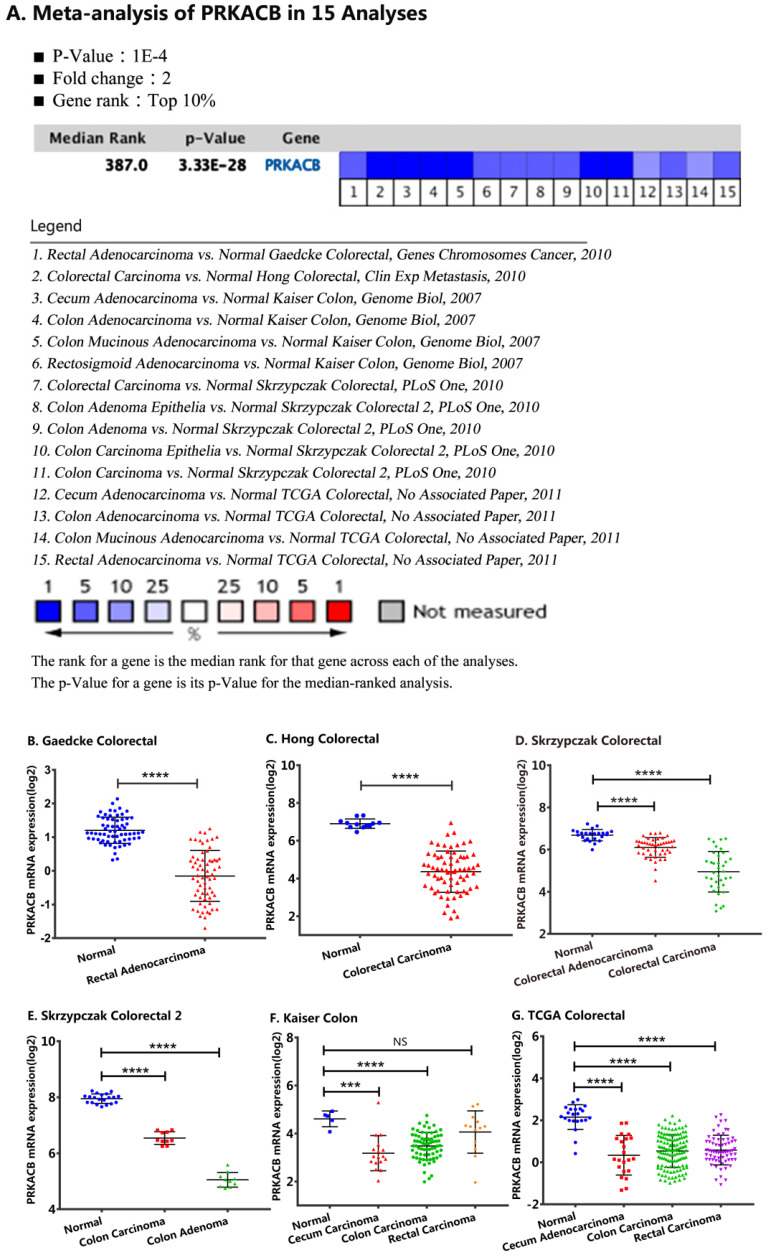
Comparison of PRKACB mRNA expression levels across 15 analyses in the Oncomine database. Meta-analysis of PRKACB expression in 15 analyses (A); PRKACB levels in Gaedcke Colorectal (B), Hong Colorectal (C), Skrzypczak Colorectal (D), Skrzypczak Colorectal 2 (E), Gaedcke Colorectal (F) and, TCGA Colorectal (G). **Note:** In the Kaiser Colon study, Colon Adenocarcinoma (n=41), Colon Mucinous Adenocarcinoma (n=13), Colon Signet Ring Cell Adenocarcinoma (n=2), Colon Small Cell Carcinoma (n=2), Rectosigmoid Adenocarcinoma (n=10), Rectosigmoid Mucinous Adenocarcinoma (n=2) were all merged into Colon Carcinoma; Rectal Adenocarcinoma (n=8), Rectal Mucinous Adenocarcinoma (n=4), Rectal Signet Ring Cell Adenocarcinoma (n=1) were merged into Rectal Carcinoma; In the TCGA Colorectal study, Colon Adenocarcinoma (n=101), Colon Mucinous Adenocarcinoma (n=22), Rectosigmoid Adenocarcinoma (n=3), Rectosigmoid Mucinous Adenocarcinoma (n=1) were merged into Colon Carcinoma; Rectal Adenocarcinoma (n=60), Rectal Mucinous Adenocarcinoma (n=6) were equally merged into Rectal Carcinoma.

For validation, we performed a meta-analysis of PRKACB expression in 15 analyses with a threshold set as p-Value ≤1E-4, fold change ≥ 2 and top 10% gene rank in the Oncomine database. As displayed in Figure 2 and compared with its value in normal tissues, PRKACB was significantly downregulated in rectal adenocarcinoma (P<0.0001, Figure 2B), rectal carcinoma (P<0.0001, Figure 2G), colon carcinoma (P<0.0001, Figure 2E-G), colon adenoma (P<0.0001, Figure 2E), colorectal carcinoma (P<0.0001, Figure 2C-D), and colorectal adenocarcinoma (P<0.0001, Figure 2D). Notwithstanding, the difference of PRKACB expression in rectal carcinoma has no value (P = 0.2021, Figure 2F). Besides, the PRKACB was also downregulated in cecal carcinoma (P<0.0005, Figure 2F) and cecal adenocarcinoma (P<0.0001, Figure 2G).

### Associations between PRKACB and survival in CRC patients

**Figure 3:**
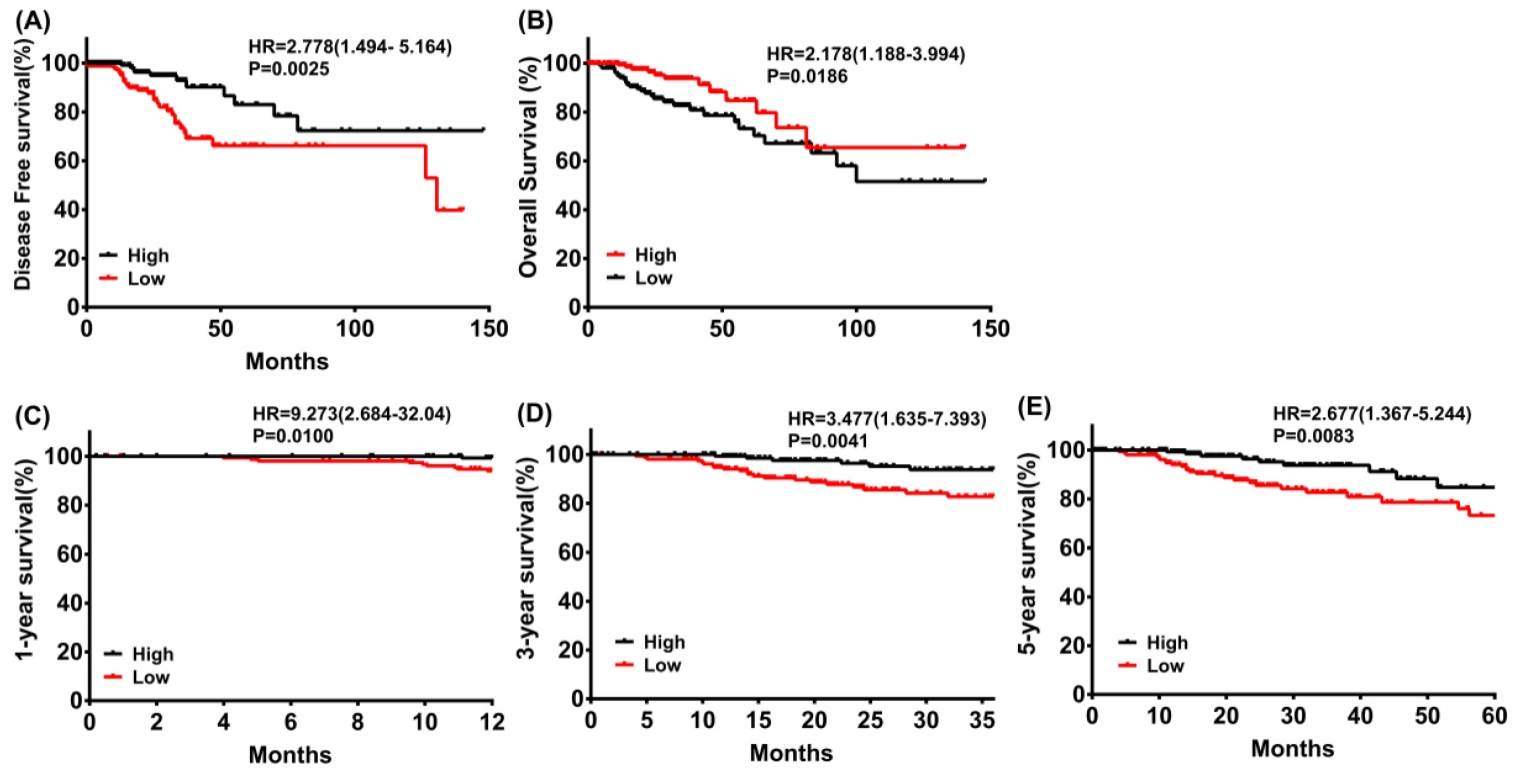
Disease-free survival (A) and overall survival (B) of CRC patients grouped by PRKACB median cutoff in TCGA database; 1-year (C), 3-year (D) and 5-year (E) overall survivals comparison between high and low PRKACB groups.

The results outlined that low expression of PRKACB in tumor tissues was considerably associated with poor disease-free survival (log rank P = 0.0025, Figure 3A) and overall survival (log rank P = 0.0186, Figure 3B) in patients with CRC. Moreover, a subgroup analysis revealed that the downregulation of PRKACB in tumor tissue was a risk factor for reduced 1 year (log rank P = 0.0100, HR = 9.273 (2.684-32.04), Figure 3C), 3 years (log rank P = 0.0041, HR = 3.477 (1.635-7.393), Figure 3D), and 5 years (log rank P = 0.0083, HR = 2.677 (1.367-5.244), Figure 3C) OS in CRC patients.

**Figure 4:**
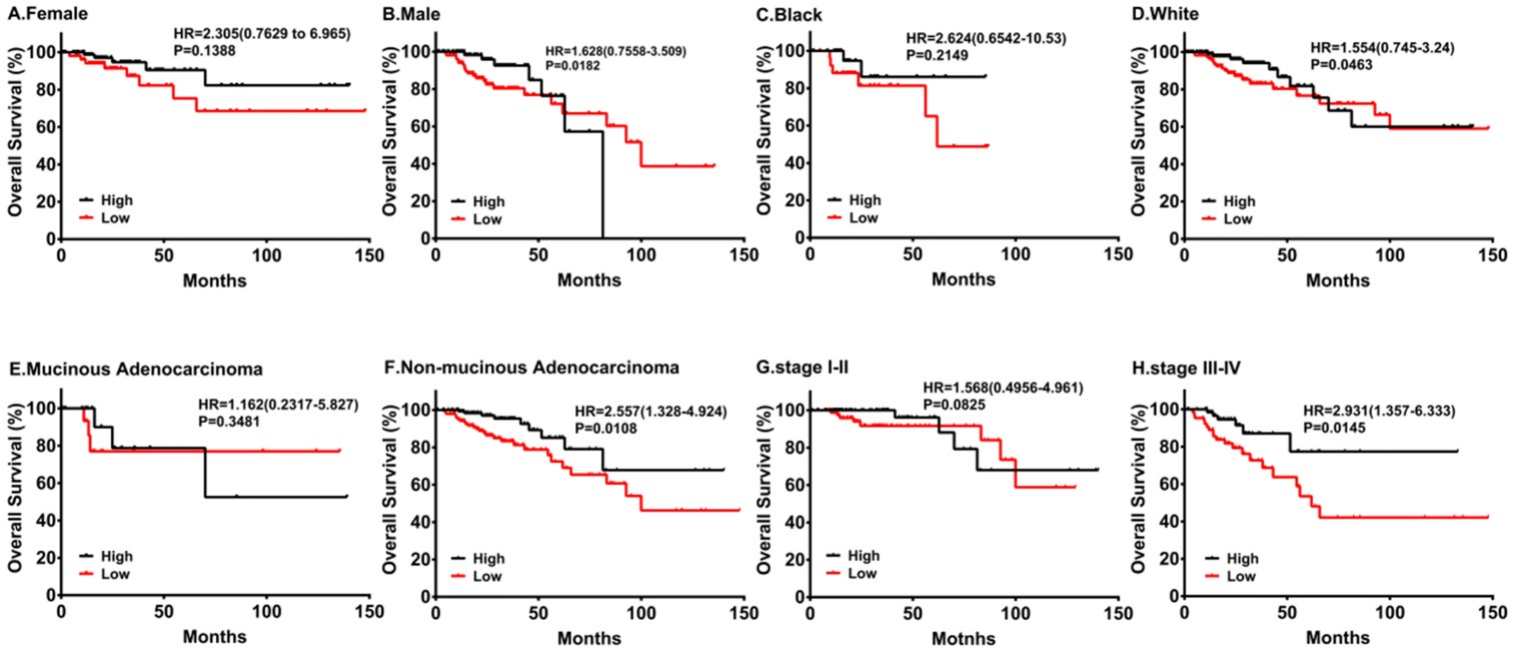
Subgroup analyses of overall survival comparison in different populations [gender (A, B), race (C, D) and cancer type (E, F)] and different stage (G, H) with PRKACB median cutoffs in CRC patients.

By the same token, we performed subgroup survival analyses in different populations. As shown in Figure 4, downregulation of PRKACB was associated with poorer survival in males and white patients, while no significant differences were found in females and black patients. Moreover, low PRKACB levels significantly contributed to worse OS in CRC patients without non-mucinous cancers (HR=2.557(1.328-4.924), log-rank P=0.0108, Figure 4E) and the down-regulation of PRKACB was observed in patients with stage III-IV colorectal cancer (HR = 2.931 (1.357-6.333), log-rank P = 0.0145, Figure 4G), but not identified as risk factors for patients with stage I-II colorectal cancer (P> 0.05, Figure 4H).

### Association between PRKACB and clinicopathological features in CRC patients

As delineated in Table 2, there are more male cases in the PRKACB low group (64.85% vs. 46.99%, P = 0.001) and PRKACB low group patients were significantly older than those in the PRKACB high group (66yr vs. 61.5yr, P = 0.022). However, BMI, tumor stage, lymph node stage, metastasis stage, AJCC stage, lymphovascular invasion, perineural invasion, vascular invasion, race category, person neoplasm status were not significantly different in PRKACB expression (P> 0.05).

### KEGG/GO biological process enrichment

**Figure 5:**
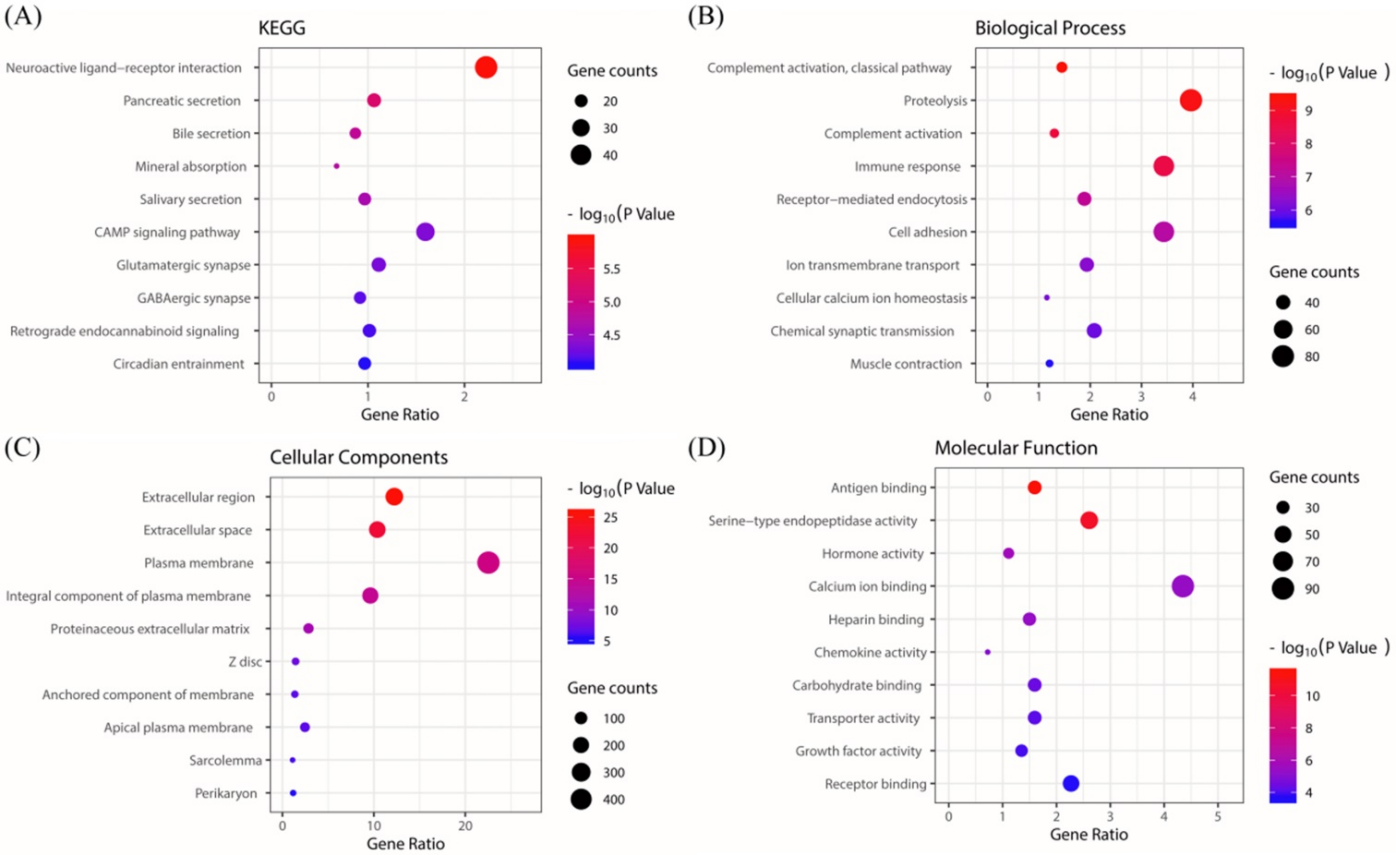
KEGG and GO biological function enrichment analyses of PRKACB related genes. KEGG signal pathway enrichment analysis (A); Biological process enrichment analysis (B), Cell component enrichment analysis (C) and molecular function enrichment analysis (D).

The KEGG pathway enrichment of PRKACB interactive genes showed that neuroactive ligand-receptor interactions, pancreatic secretions, bile secretions, mineral absorption, salivary secretions, cAMP signaling pathways, glutamatergic synapse, GABAergic synapses, retrograde endocannabinoid signaling, circadian entrainment, etc. were the most enriched pathways (Figure 5A). Additionally, GO analysis results proved that PRKACB-related genes were significantly enriched in the complement activation classical pathway, proteolysis, complement activation, etc. at BP levels; extracellular region, extracellular space, plasma membrane, etc. at CC levels and antigen-binding, serine-type endopeptidase activity and hormone activity at MF levels (Figure 5B-D).

### Protein-protein interaction (PPI) network construction

A total of 127 genes were filtered into the target genes PPI network complex, containing 100 nodes and 654 edges, 10 hub Genes (PRKACB, ATP2B2, MAPT, PHLPP2, ABCCB, GRIN2A, MYLK, GRIA1, BCHE, ADCY2) were screened according to Cytoscape 3.7.1 and its plug-in (Ranking degree of cytoHubba).

### Gene Co-expression Network Analysis

**Figure 7:**
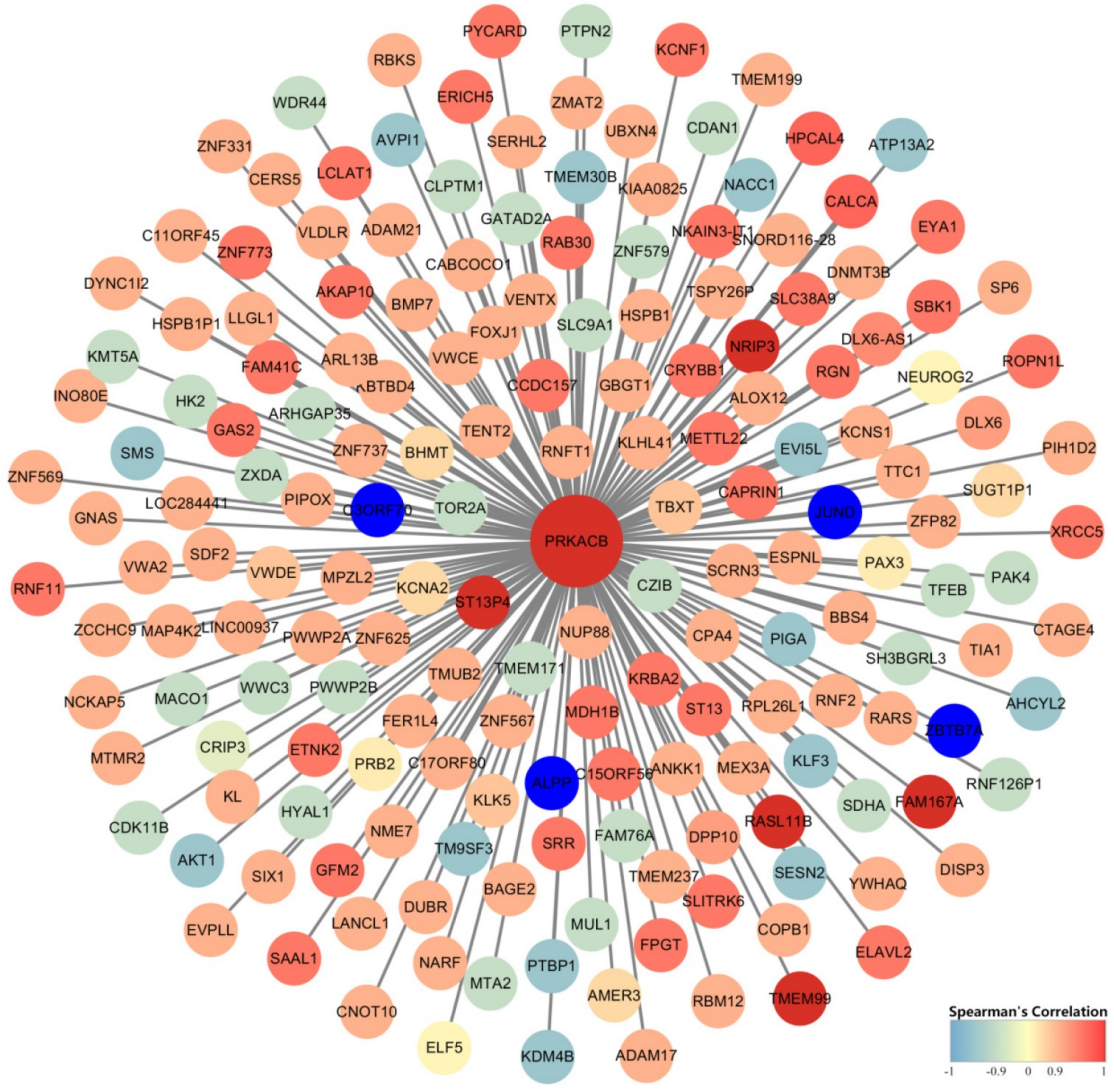
Construction of gene co-expression networks. Blue represents genes that are negatively related to PRKACB, and red points out genes that are positively related to PRKACB. The darker the color, the stronger the correlation.

Identification of PRKACB co-expressed genes was completed by the cBioPortal database's online analysis function. A total of 182 genes with Spearman correlation coefficients greater than 0.9 (highly correlated) were selected and visualized via Cytoscape (version 3.7.1) (Figure 7). We found that FAM167A, NRIP3, RASL11B, ST13P4, TMEM99 completely correlated with PRKACB (Spearman's Correlation = 1) whereas ALPP, C3ORF70, JUND, and ZBTB7A were completely negative correlated with PRKACB (Spearman's Correlation = -1).

## Discussion

Over these past years, more and more data indicate that PRKACB is involved in the cancerization process of malignant tumors in different systems. PRKACB mutations and gene fusion of PRKACB can lead to adrenal, bile duct, liver, and pancreatic cancers 12,13, meanwhile down-regulation of PRKACB expression may be associated with poor survival in patients with non-small cell lung cancer. Makondi et al. found that targeting PRKACB may increase the responsiveness of colorectal tumors to irinotecan treatment 28, and Feng et al. also found pathological expressions of PRKACB in human colorectal cancer tissues infected with nucleobacteria 29. Consistent with previous studies 30, our study showed that PRKACB had a significant discrepancy of expression in normal and tumor tissues of colorectal cancer, and we also depicted that down-regulation of PRKACB expression is a risk factor for declining 1, 3, and 5-year survival rates in patients with CRC. This was equally associated with poor patient-free survival and overall survival. Consequently, we can confirm that PRKACB plays an important role in patients with colorectal cancer and affects the prognosis of patients.

Gene co-expression network analysis established that PRKACB had a completely positive correlation with FAM167A, NRIP3, RASL11B, ST13P4, TMEM99, and a completely negative correlation with ALPP, C3ORF70, JUND, and ZBTB7A. It has been confirmed by studies that NRIP3 and RASL11B play a role in suppressing cancer proliferation in breast cancer and renal cell carcinoma 31,32. Meanwhile ALPP, JUND, ZBTB7A in gastric cancer, prostate cancer, breast cancer 33-35 and other cancers promote the progress of cancer. Thence, we can boldly speculate that PRKACB plays a synergistic role with these tumor suppressors in inhibiting tumor growth. On the contrary, oncogenes antagonize the tumor-suppressive effect of PRKACB.

Many studies have confirmed that microRNAs can regulate the expression of PRKACB 29,36-38, affect cell proliferation, adhesion, and metastasis, and eventually bring about the development of tumors. Also, studies have found that antisense oligodeoxynucleotides targeted to protein kinase subunits induce growth arrest, apoptosis, and differentiation in a variety of cancer cell lines both *in vitro* and *in vivo* 39. Similarly, we were able to regulate the development of colon cancer by targeting PRKACB, providing an important theoretical basis for the development of colon cancer chemotherapeutic drugs and new anti-EMT therapies.

The protein kinase cAMP-dependent catalytic subunit β (PRKACB) encoding the cAMP-dependent protein kinase catalytic subunit β (PRKACB) is a member of the serine/threonine protein kinase family 7. Activation of PKA catalytic activity is initiated by any signal causing an increase in intracellular cAMP concentration. The traditional view of PKA activation is that two cAMP molecules bind to each R subunit, causing a conformational change in the R subunit dimer, and the C subunits are released and become catalytically active through exposure of their active sites 40. PKs catalyze the transfer of phosphate groups onto Ser, Thr, or Tyr residues of target proteins. Phosphorylation of substrates represents a key regulatory mechanism in all eukaryotic cells^41^ and the various PKs target different substrates with a multitude of biological effects. In addition, studies have shown that PKA-mediated tamoxifen resistance in breast cancer is caused by 305 serine (S305) phosphorylation of Erα 42. Similarly, phosphorylation of PRKACB has also been found in neurological diseases 37. From a physiologic aspect, PRKACB may mainly regulate protein phosphorylation/dephosphorylation through the cAMP pathway. Unfortunately, we are yet to conduct experimental studies exploring the potential carcinogenic mechanism of PRKACB in the development of liver cancer. Even after taking into account previous reports, we are pragmatic on suggesting the hypothesis that low expression of PRKACB leads to poorer prognoses in CRC patients.

## Figures and Tables

**Figure 6:**
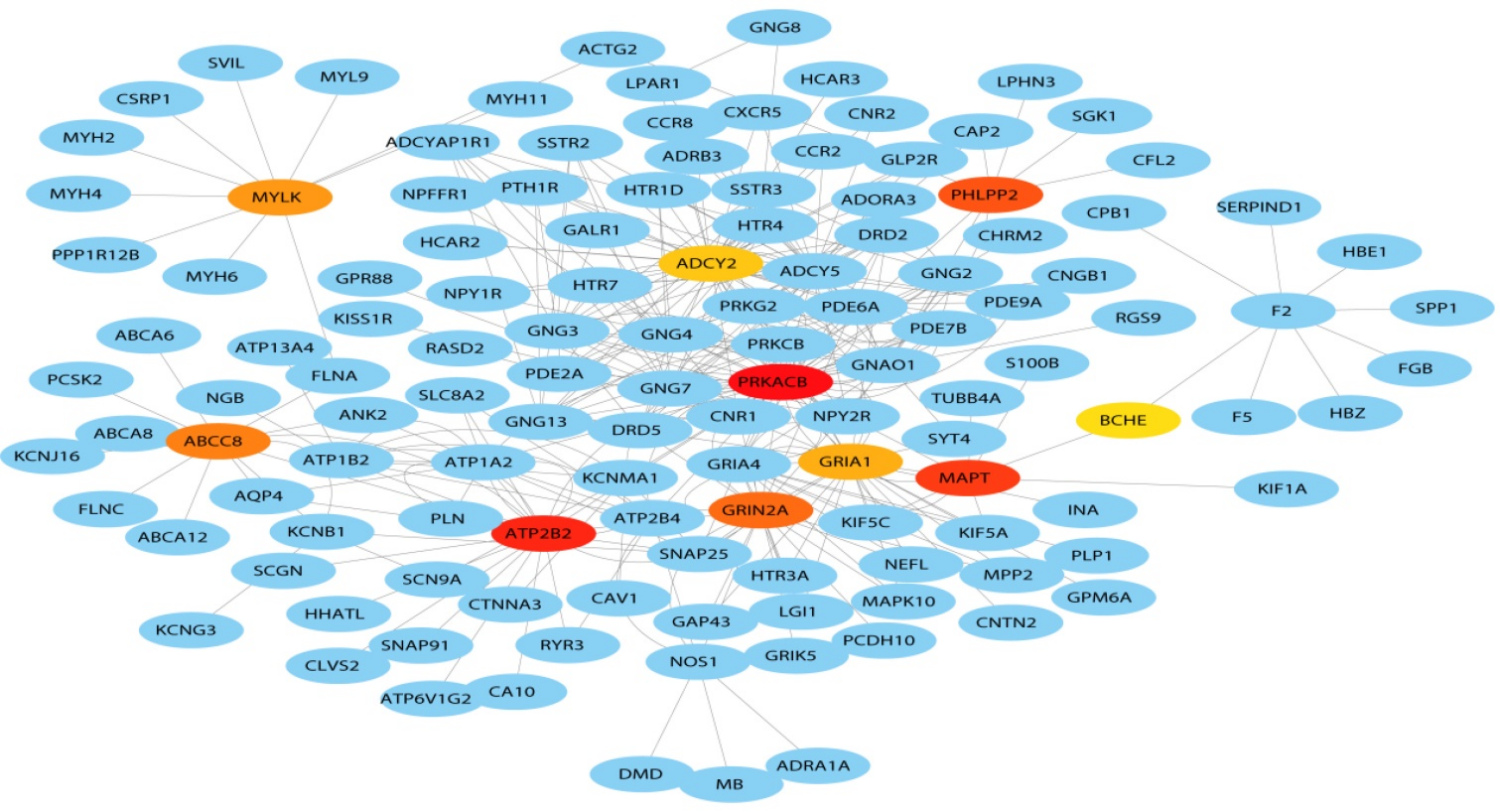
Hub genes of the PPI network. The darker the color, the bigger the degrees.

**Table 1:** Details of GEO series included in this analysis

GEO series	Contributor(s), Year	Tumor	Non-tumor	Platform
GSE110225	Vlachavas E, 2018	30	30	[HG-U133A] Affymetrix Human Genome U133A Array[HG-U133_Plus_2] Affymetrix Human Genome U133 Plus 2.0 Array
GSE32323	Ahmed K et al., 2012	17	17	[HG-U133_Plus_2] Affymetrix Human Genome U133 Plus 2.0 Array
GSE44076	Solé X et al., 2014	98	98	[HG-U219] Affymetrix Human Genome U219 Array
GSE9348	Hong Y et al., 2010	70	12	[HG-U133_Plus_2] Affymetrix Human Genome U133 Plus 2.0 Array
GSE41328	Lin G et al., 2012	5	5	[HG-U133_Plus_2] Affymetrix Human Genome U133 Plus 2.0 Array
GSE21510	Tsukamoto S, 2011	19	25	[HG-U133_Plus_2] Affymetrix Human Genome U133 Plus 2.0 Array
GSE68468	NA, 2015	44	44	[HG-U133A] Affymetrix Human Genome U133A Array

**Table 2:** Characteristics of CRC patients between PRKACB high and low groups

		High groups (n=166)	Low groups (n=165)	*P*
Gender^✱^	Male (n=185)	78	107	0.001
	Female (n=146)	88	58	
Age, median(IQR), years^✱^		61.5(21.75)	66(17)	0.022
	≤60 (n=139)	80	59	
	>60 (n=192)	86	106	
BMI, kg/m2	<18.5 (n=4)	1	3	0.836
	18.5-24.99 (n=86)	44	42	
	25-29.99 (n=104)	53	51	
	>29.99 (n=60)	29	31	
Tumor Stage	T1 (n=10)	4	6	0.849
	T2 (n=49)	24	25	
	T3 (n=231)	115	116	
	T4 (n=40)	22	18	
	Tis (n=1)	1	0	
Lymph Node Stage	N0 (n=188)	91	97	0.513
	N1 (n=87)	46	41	
	N2 (n=54)	29	25	
	NX (n=2)	0	2	
Metastasis Stage	M0 (n=228)	111	117	0.143
	M1 (n=40)	26	14	
	MX (n=59)	28	31	
AJCC stage	I (n=52)	25	27	0.213
	II (n=125)	61	64	
	III (n=99)	47	52	
	IV (n=41)	27	14	
Lymphovascular invasion	YES (n=89)	43	46	0.910
	NO (n=206)	101	105	
Perineural Invasion	YES (n=51)	30	21	0.196
	NO (n=153)	74	79	
Vascular invasion	YES (n=61)	31	30	0.674
	NO (n=226)	108	118	
Race Category	WHITE (n=232)	112	120	0.479
	BLACK OR AFRICAN AMERICAN (n=56)	28	28	
	ASIAN (n=12)	8	4	
	AMERICAN INDIAN OR ALASKA NATIVE (n=1)	1	0	
Person Neoplasm Status	TUMOR FREE (n=221)	115	106	0.845
	WITH TUMOR (n=71)	36	35	
